# A Multidimensional Assessment of a Novel Adaptive Versus Traditional Passive Ankle Sprain Protection Systems

**DOI:** 10.1177/03635465221146294

**Published:** 2023-02-03

**Authors:** Steffen Willwacher, Anna Bruder, Johanna Robbin, Jakob Kruppa, Patrick Mai

**Affiliations:** † Department of Mechanical and Process Engineering, Offenburg University of Applied Sciences, Offenburg, Germany; ‡ Institute of Biomechanics and Orthopaedics, German Sport University Cologne, Cologne, Germany; Investigation performed at Offenburg University of Applied Sciences, Offenburg, Germany; biomechanical data collection performed in the laboratory of the Institute of Functional Diagnostics, Cologne, Germany

**Keywords:** ankle sprain, injury, protective equipment, adaptive technology, inversion

## Abstract

**Background:**

Ankle braces aim to reduce lateral ankle sprains. Next to protection, factors influencing user compliance, such as sports performance, motion restriction, and users’ perceptions, are relevant for user compliance and thus injury prevention. Novel adaptive protection systems claim to change their mechanical behavior based on the intensity of motion (eg, the inversion velocity), unlike traditional passive concepts of ankle bracing.

**Purpose:**

To compare the performance of a novel adaptive brace with 2 passive ankle braces while considering protection, sports performance, freedom of motion, and subjective perception.

**Study Design:**

Controlled laboratory study.

**Methods:**

The authors analyzed 1 adaptive and 2 passive (one lace-up and one rigid brace) ankle braces, worn in a low-cut, indoor sports shoe, which was also the no-brace reference condition. We performed material testing using an artificial ankle joint system at high and low inversion velocities. Further, 20 male, young, healthy team sports athletes were analyzed using 3-dimensional motion analysis in sports-related movements to address protection, sports performance, and active range of motion dimensions. Participants rated subjective comfort, stability, and restriction experienced when using the products.

**Results:**

Subjective stability rating was not different between the adaptive and passive systems. The rigid brace was superior in restricting peak inversion during the biomechanical testing compared with the passive braces. However, in the material test, the adaptive brace increased its stiffness by approximately 400% during the fast compared with the slow inversion velocities, demonstrating its adaptive behavior and similar stiffness values to passive braces. We identified minor differences in sports performance tasks. The adaptive brace improved active ankle range of motion and subjective comfort and restriction ratings.

**Conclusion:**

The adaptive brace offered similar protective effects in high-velocity inversion situations to those of the passive braces while improving range of motion, comfort, and restriction rating during noninjurious motions.

**Clinical Relevance:**

Protection systems are only effective when used. Compared with traditional passive ankle brace technologies, the novel adaptive brace might increase user compliance by improving comfort and freedom of movement while offering similar protection in injurious situations.

Ankle sprains are among the most common traumatic injuries in athletes,^5^ with the highest incidences observed in indoor and court sports.^4^ Ankle sprains represent 10% to 28% of all sports-related injuries, and approximately 73% of competitive and recreational athletes experience recurrent ankle sprains.^6,22^ Data captured from ankle sprain injuries suggest that most ankle sprains occur at ankle inversion angles >30° and peak ankle inversion velocities >500 deg/s.^10^

Successful prevention of ankle injury and reinjury includes neuromuscular training with passive protection systems (eg, ankle braces).^16^ Ankle brace design should protect the joint from excessive motions. However, clinical experience suggests that poor comfort (caused, eg, by poor fit, restricted motion, or pressure peaks due to rigid parts) or a potential reduction of sports performance (eg, due to restricted joint movement) might lead to noncompliance of athletes in use of braces for ankle sprain prevention.^7,13^ Therefore, we propose to test the preventive effects of ankle protection technology (including ankle braces) in 4 domains:

1. The protection domain (ie, reduction of peak ankle inversion angles during sudden inversion or supination motions): these motions can be induced on tilt platforms or during change of direction tasks. However, because of ethical restrictions, peak ankle angles need to stay within physiological (ie, noninjurious) ranges during biomechanical testing. Therefore, the true protective potential of ankle protection technology can only be estimated from these interventions and should be supplemented by systematic material testing using artificial ankle joints or cadaveric specimens. This approach allows for systematic variation of loading parameters (eg, angular velocities, ankle ranges of motion). The passive nature of these tests is justified by the lack of active muscular control of ankle inversion motion typically reported in unexpected sudden inversion motions.^8^
2. The sports performance domain: it is unlikely that competitive athletes will sacrifice their sports performance to prevent injuries. Team sports performance can be quantified for acceleration, change of direction, or jumping tasks, which frequently occur during football, basketball, or handball.
3. Subjective comfort and stability rating: subjective perception of the stabilizing effect of an ankle protection technology with a high comfort rating would likely increase user compliance.
4. Freedom of movement during nonexcessive ankle ranges of motion: reducing the physiological degrees of freedom of the ankle joint would likely reduce comfort perception and sports performance.

Optimizing the trade-off between the 4 domains might enhance the preventive effect of an ankle protection device.

Passive ankle protection systems, including ankle braces, have frequently been assessed within the literature.^3,17^ However, in most of these studies, the 4 mentioned domains have only partially been addressed. Further, almost all studies considered passive ankle braces. Recent advances have allowed for the creation of an adaptive protection behavior of sports protection technologies. Although it was elegantly shown that an ankle brace incorporating such an adaptive protection technology protects the ankle against sudden inversion motions compared with a placebo control condition,^1^ an assessment of adaptive ankle braces against traditional passive ankle braces while considering the 4 domains of ankle protection has not yet been performed.

Therefore, the purpose of the present study was to evaluate the performance of a novel adaptive ankle brace compared with traditional passive ankle brace concepts while considering the highlighted domains of ankle protection technology. We hypothesized that the adaptive ankle brace would restrict movement less during slower, nonexcessive ankle motions. Because of the adaptive stiffening of the novel ankle brace during high angular velocities, we hypothesized a similar protective effect of the adaptive brace to those of the passive braces during sudden inversion motions. Because of the greater freedom to move, we further hypothesized an improved sports performance and subjective comfort rating of athletes using the adaptive compared with the passive ankle braces.

## Methods

### Participants

Twenty male, regional-level team sports (soccer, handball, basketball) athletes (age, 24.3 ± 3.4 years; height, 1.84 ± 0.05 m; weight, 81.3 ± 7.4 kg; 16 right-leg dominant, 4 left-leg dominant) participated in the study. Based on an a priori power analysis, 20 participants was considered sufficient to identify a difference of 4° in maximal inversion angle between 2 conditions (alpha, .05; power, 0.8; SD, 6°^1^). Participants were injury-free in the 12 months preceding data collection and signed written informed consent before their participation. Only 1 participant had undergone anterior cruciate ligament reconstruction surgery, approximately 3 years before data collection. The other participants had not undergone lower extremity surgery. Four participants reported previous ankle sprain injuries >12 months before data collection (ranging from 2 to 13 years before data collection; 2 participants sprained their ankles on the left and 2 on the right side). All methods used in the study had been approved by the research ethics committee of the university.

### Experimental Protocol

We analyzed 1 adaptive brace (Sportomedix Malleo Fast Protect, with Betterguards adaptive technology) and 2 passive ankle braces (lace-up, Basko; rigid brace, T2 Active Ankle). The Betterguards adaptive technology consists of a semiflexible mini-piston embedded in an adaptor element crossing the lateral side of the ankle, including a valve. This valve allows fluid to pass within the piston while extending at physiological movement velocities. In critical movement velocities, the valve closes within milliseconds because of fluid dynamic drag forces and inhibits the further extension of the mini-piston, resulting in a limited range of motion. Participants wore all braces in the identical low-cut indoor sports shoe (Mizuno Wave Mirage 3), which also served as the no-brace reference condition.

To address the 4 domains of effective ankle protection, we developed a test battery of different motion tasks. Participants wore the braces on both the left and the right ankle joints during each task and brace condition. We captured joint kinematics with a 3-dimensional (3D) motion capture system (200 Hz, 12 Miqus M3 cameras; Qualisys AB) synchronized with ground-embedded force platforms. We attached spherical retroreflective markers (diameter, 13 mm) to 38 bony landmarks.^14,15,21^ We attached foot markers at the corresponding positions on the shoe. We filtered all marker trajectories with a recursive, fourth-order digital Butterworth filter (cutoff frequency, 10 Hz).^11^ A 3D rigid body model of the pelvis and the lower extremities, consisting of 9 rigid body segments (including a rearfoot and a forefoot segment), was used to calculate 3D joint angles at the hip, knee, and ankle joint.^19,20^ Specifically, the ankle joint movement was defined as the movement of the rearfoot segment relative to the shank segment. Joint angles were extracted as Cardan angles from the rotation matrix between rearfoot and shank segments using a flexion-extension, inversion-eversion, internal-external rotation sequence of rotation.

### Protection Domain

To test the protective effects of the braces, we used a tilting platform that induced a combination of tilt around an antero-posterior (30° platform tilt) and mediolateral (10° platform tilt) axis at an angular velocity of 440 deg/s unexpectedly.^2^ Thus, the platform provoked sudden inversion and plantarflexion motion of the ankle joint complex. While the tilting was induced on the right foot, the left foot was supported by 3 one-dimensional force sensors, measuring vertical ground-reaction forces (GRFs). Using these force measurements via real-time feedback, we controlled the weight distribution between legs (80% on the tilted side). We further controlled a relaxed standing position by real-time monitoring surface electromyography (EMG) of the peroneus longus and tibialis anterior muscles using a wireless EMG system (2000 Hz; Aktos; Myon AG).

**Figure 1.**
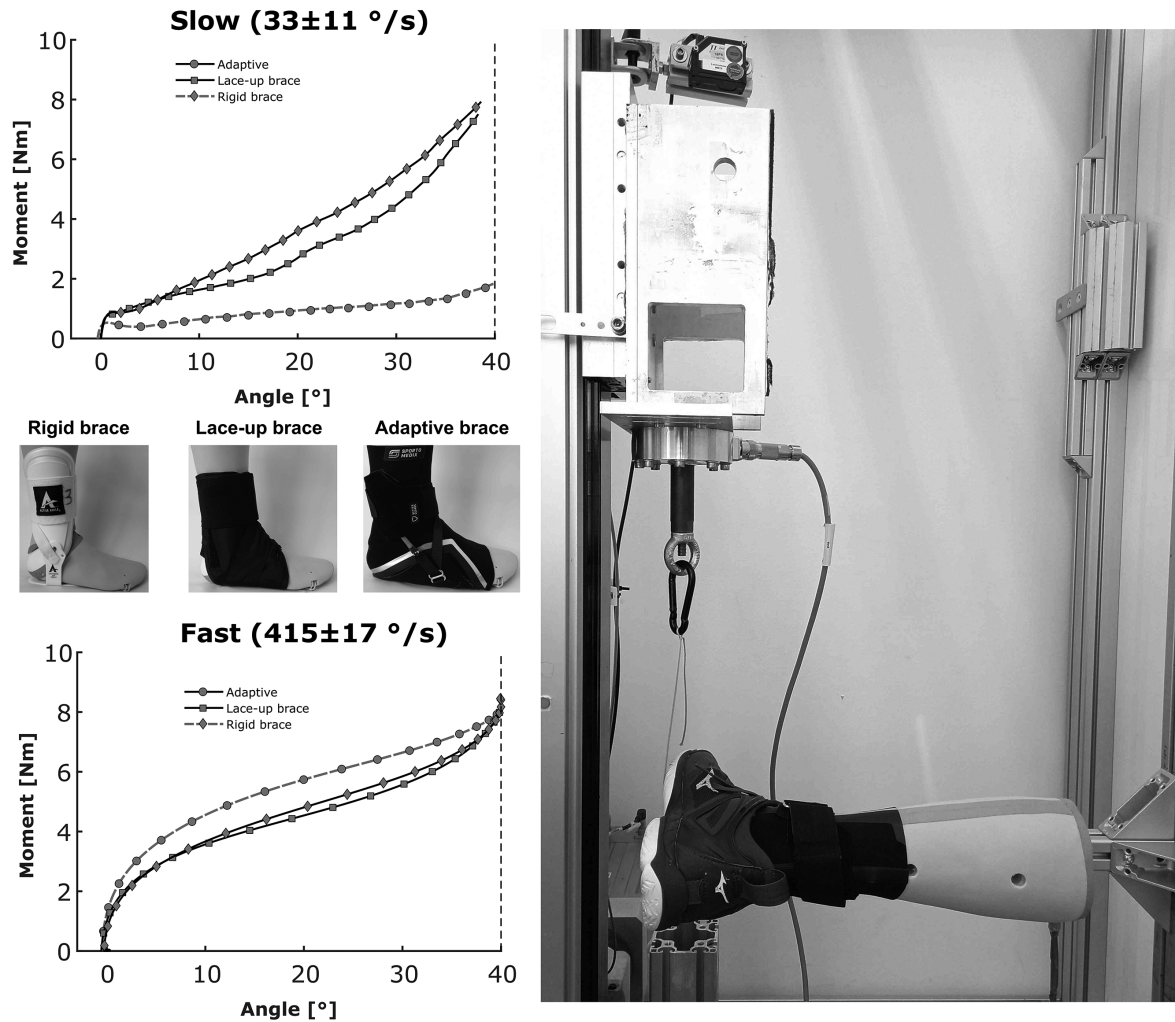
Mechanical tests performed with the artificial ankle (representing the right lower leg and foot) on the different brace conditions. Inversion is induced by pulling the rope connected to the lateral aspect of the foot upward. All braces were tested with the same shoe as during the biomechanical testing. Results represent the mean of 3 trials per condition.

For ethical reasons, we could not test the protective capacity of the ankle braces at tilt angles >30° and very high tilt velocities. Because ankle sprains occur more often in these more extreme test scenarios,^10^ we developed a mechanical test procedure with an artificial lower leg and foot (Figure 1). In this artificial device, the lower leg and foot are connected by a joint in which the axis is motivated by the natural tilt of the human subtalar joint axis. We performed mechanical testing at 33 ± 11 deg/s and 415 ± 17 deg/s to simulate slow and fast ankle inversion motions, respectively. Inversion movements up to 40° were induced by a rope that pulled the lateral part of the foot upward (into combined inversion and plantarflexion) (Figure 1). We quantified the external joint moment by multiplying the resultant force applied by the material testing machine within the pulling rope with the respective moment arm to the ankle joint center. Joint angles were measured using an embedded electrogoniometer. All measurements were sampled with a frequency of 2000 Hz. We subtracted the joint moment that occurred due to the inherent friction within the apparatus by performing a measurement without any shoes or orthoses. From the measurements, we calculated mean (0°-40° inversion) stiffness as the change in external joint moment divided by the change in joint angle within the respective interval.

We further analyzed ankle joint kinematics during a maximum effort 90° change of direction task. The participants performed these cutting maneuvers from a 4-step approach and were instructed to perform the task with maximal intensity. The final biomechanical task for analyzing joint protection was repeated side-shuffle motions from the left to the right leg. We instructed the participants to vary the intensity of task execution within each of the 3 performed trials. We quantified the mean horizontal GRF applied within each ground contact. We then extracted only those ground contacts in which the mean horizontal GRF was within 70% to 90% of the maximum value obtained in any ground contact in any brace condition. With this approach, we could compare ankle joint kinematics between conditions for the same relative task intensity.

### Sports Performance Domain

To compare the effects on sports performance between brace conditions, participants performed a linear acceleration task, a vertical countermovement jump (CMJ), a 90° change of direction, and a single-leg side-hopping task over a distance of 30 cm with maximum effort.

The linear acceleration task was performed from a standing start position in front of a floor-mounted force platform (2000 Hz, 0.9 × 0.6 m; AMTI).^19^ We analyzed the GRFs of the first contact after the onset of the motion. We divided the change in running velocity (achieved through integrating the body mass–normalized horizontal GRF component) by ground contact time to achieve the mean horizontal acceleration as our performance criterion during this task. For the CMJ, we quantified performance via the achieved jump height. We did not find any statistically significant differences between brace conditions for entry and exit center of mass velocity (estimated via the velocity of the center of the pelvis), as well as for the cutting angle. Therefore, we quantified performance during the cutting task through the execution, that is, ground contact time. The pelvis velocity was determined by numerical differentiation of the horizontal components of the midpoint of the pelvis segment (ie, the midpoint between the 4 pelvis markers). The actual change of direction angle during the cutting task was determined using the angles between the horizontal components of the velocity vectors of the pelvis markers averaged over 5 data frames before and after ground contact. Performance during the side-hopping task was evaluated by the execution time needed to perform 5 right-left single-leg jumps.

### Subjective Comfort and Stability Perception Domain

We asked the participants for their subjective comfort and stability rating of the analyzed braces on a 10-cm visual analog scale (VAS). For comfort and stability ratings, higher VAS values represent a more comfortable or more stable condition, while for the rating of perceived restriction, a higher value refers to less restriction.

### Freedom of Motion Domain

We assessed ankle range of movement in the frontal plane during a sitting, low-speed ankle inversion-eversion movement. The participants were advised to follow a metronome set to 20 beats per minute (0.33 Hz) and achieve maximal active eversion and inversion excursions. The maximum range of movement achieved in the frontal plane during 10 motion cycles quantified freedom of motion.

### Statistical Analysis

We present all parameters as group means (and standard deviations). We applied 1-factor (brace condition) repeated-measures analysis of variance to identify the ankle brace condition main effects for our parameters of interest. In the case of a brace condition main effect, we performed pairwise comparisons between brace conditions using dependent-sample *t* tests. Because of the explorative nature of the study, we did not correct for multiple comparisons when analyzing differences between individual braces. Furthermore, Cohen *d* effect sizes were calculated to evaluate the strength of the observed effects for each brace compared with the no-brace condition, using the equation

(1) $$d=\frac{M_{\mathrm{Brace}}-M_{\mathrm{No}\;\mathrm{Brace}}}{\sqrt{\frac{\left({\mathrm{SD}}_{\mathrm{Brace}}^{2}+{\mathrm{SD}}_{\mathrm{No}\;\mathrm{Brace}}^{2}\right)}{2}}}$$

with *M_Brace_* and *M_No Brace_* being the mean values of a brace condition and the no-brace condition, respectively. *SD_Brace_* and *SD_No Brace_* are the standard deviations of a brace condition and the no-brace condition, respectively. All analyses were performed using MATLAB statistics and machine learning toolbox (R2019b; The MathWorks Inc). The significance level was set to an α level of 5% (*P* < .05).

## Results

### Protection Domain

**Figure 2.**
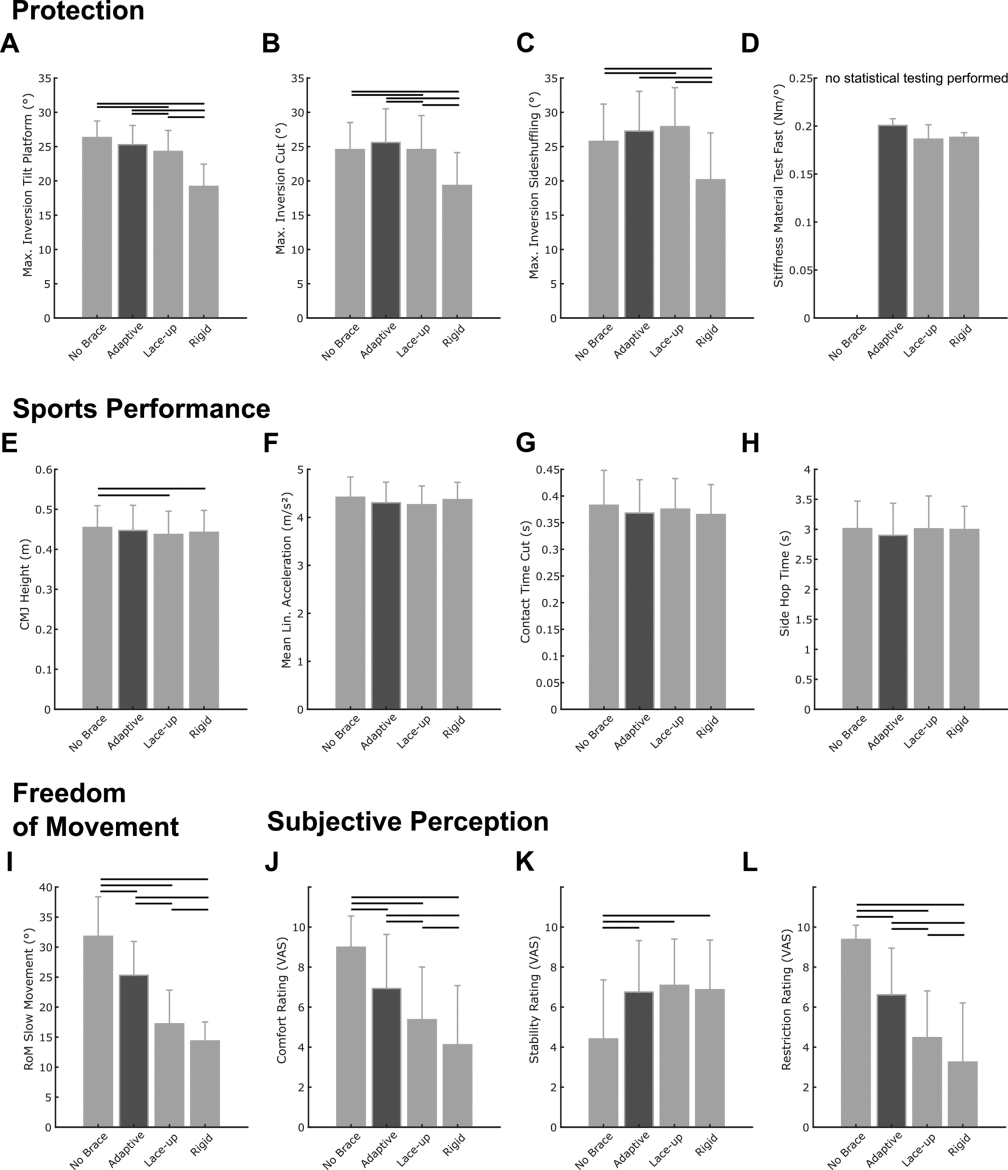
Summary of differences observed for key biomechanical parameters within the 4 domains of ankle sprain protection (A to D: Protection dimension; E to H: Sports performance dimension; I: Freedom of movement dimension; J to L: Subjective Perception dimension). Numeric data of these results are summarized in Appendix Table A1 (available online). Horizontal lines indicate a statistically significant difference between 2 conditions (*P* < .05). Mean Lin., average linear; CMJ, countermovement jump; RoM, range of motion; VAS, visual analog scale.

We observed significant main effects of the ankle brace condition for all biomechanical parameters related to ankle joint protection (Figure 2, A-C; see also Appendix Table A1, available in the online version of this article). Post hoc analyses revealed that the adaptive brace decreased peak inversion during induced sudden inversion and plantarflexion motions on the tilt platform (–4.0% to baseline; *P* = .056; *d* = 0.40) less than the lace-up (–7.7% to baseline; *P* = .003; *d* = 0.74) and rigid (–27.1%; *P* < .001; *d* = 2.52) braces (Figure 2A; see also Appendix Table A1, available online). The difference to the lace-up brace was, on average, 1.0° (*P* = .039; *d* = 0.37) (see Appendix Table A1, available online).

In the change of direction and side-shuffling tasks, the rigid brace achieved significant reductions in peak inversion compared with all other conditions (see Appendix Table A1, available online). The adaptive brace or lace-up brace did not result in significant reductions of peak inversion in these neuromuscular controlled motion tasks.

Interestingly, the participants reached higher absolute peak inversion angles during the preplanned change of direction and side-shuffling tasks compared with the unexpected tilt platform inversion (Figure 2, B and C; see also Appendix Table A1, available online).

The material testing with the artificial ankle joint at 2 different angular velocities revealed the adaptive behavior of the adaptive brace. Although its stiffness remained relatively low during the slow inversion motion (Figure 1; see also Appendix Table A1, available online), its stiffness increased comparable with those of the lace-up and rigid braces, respectively, during the fast inversion motion (Figures 1 and 2D; see also Appendix Table A1, available online).

### Sports Performance Domain

We identified significant main effects of ankle brace condition for the parameters CMJ height and ground contact time during the change of direction task (Figure 2, E and G; see also Appendix Table A1, available online). CMJ heights were reduced for the lace-up (–3.8% to baseline; *P* = .006; *d* = 0.31) and rigid (–2.7% to baseline; *P* = .017; *d* = 0.22) braces, while the difference for the adaptive brace (–1.7% to baseline; *P* = .072; *d* = 0.13) compared with the no-brace condition did not reach the level of significance (Figure 2E; see also Appendix Table A1, available online). We observed no significant main effect of ankle brace conditions on linear acceleration performance (see Appendix Table A1, available online).

Because we did not find any significant differences between conditions regarding entry and exit velocity and the actual angle of the change in direction (see Appendix Table A1, available online), sports performance during the cutting maneuver can be quantified via the execution (ie, ground contact) time. Here, we found no significant main effect of ankle brace conditions (Figure 2G; see also Appendix Table A1, available online).

We could not identify significant differences in side-hop execution times between brace conditions (Figure 2H; see also Appendix Table A1, available online).

### Subjective Comfort and Stability Perception Domain

We observed significant main effects of ankle brace condition for all subjectively rated parameters (see Appendix Table A1, available online). The participants rated better comfort and reported that they felt less restricted when wearing the adaptive brace compared with the 2 passive braces (Figure 2, J and L; see also Appendix Table A1, available online). Each of the braces improved the stability rating of the participants, and there was no significant difference between products regarding the stability rating (Figure 2K; see also Appendix Table A1, available online).

### Freedom of Motion Domain

Compared with the no-brace baseline condition, the adaptive brace reduced the frontal plane ankle range of movement less (–20.4%; *P* < .001; *d* = 1.06) than passive ankle braces (lace-up: –45.8%; *P* < .001; *d* = 2.39; rigid brace: –54.8%; *P* < .001; *d* = 3.57) (Figure 2I; see also Appendix Table A1, available online).

## Discussion

The purpose of the present study was to evaluate the performance of a novel adaptive ankle brace compared with traditional passive ankle brace concepts while considering different domains relevant to the user compliance of ankle protection systems.

The results of the material testing at slow and high inversion velocities confirmed the adaptivity of the adaptive brace system (Figure 1). We observed a 400% increase in stiffness created during the fast simulated inversion compared with the slow simulated inversion. The traditional brace concepts did not show this adaptive behavior and showed similar results in the slow and fast supination conditions. The adaptive brace achieved slightly higher stiffness values than the traditional braces in the fast condition. Further, the adaptive brace created higher resisting moments for most of the range of motion covered during the material test (Figure 1).

However, during the biomechanical testing, the adaptive brace limited peak ankle inversion less than did the rigid brace, with peak inversion values similar to those of the lace-up brace within the different sports-specific motions analyzed. The rigid brace reduced peak inversion most substantially during these movements, with reductions between 5.3° and 7.2° compared with the no-brace baseline.

**Figure 3.**
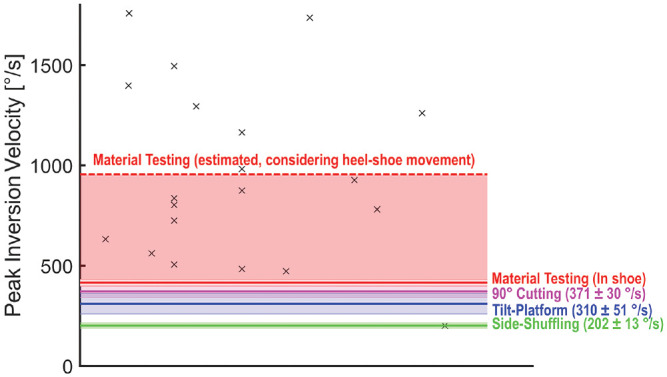
Comparison of peak inversion velocities. Black crosses indicate individual data of ankle injuries that have occurred in either a laboratory or a real game play situation, as summarized by Lysdal et al.^10^ Continuous horizontal lines indicate peak inversion velocities measured during the biomechanical and material testing. The colored areas highlight either values of ±1 SD around the mean values between orthotic conditions (actual mean ± SD values in parentheses) or the area between the actually measured and estimated peak inversion velocity considering heel-shoe movement in the material testing condition. Because angular velocity measurements during the material testing did not consider the overestimation of rearfoot motion due to marker placement on the heel cap of the shoe, we further estimated a comparable peak inversion velocity for the material testing based on the overestimation factor determined by Reinschmidt et al^12^ (factor, 2.3).

The contradiction between material and biomechanical test results might be resolved when considering the inversion velocities in the different testing situations. Figure 3 highlights the mean peak inversion velocities observed during our material and biomechanical testing in relation to data from individuals who have experienced an ankle inversion injury, summarized in a recent review article.^10^ However, when comparing the peak inversion velocities observed during our material and biomechanical testing, the different methods of measuring ankle inversion need to be considered. During the biomechanical testing, in order not to destroy the integrity of the heel cap and ankle braces, we had to place the markers on the heel cap of our baseline shoe. However, this approach is known to overestimate rearfoot motion up to 2.3-fold, on average.^12^ On the other hand, inversion was measured directly using an electrogoniometer integrated into the artificial ankle joint during the material testing. This situation represents the direct measurement of foot motion within a shoe/orthotic condition. To compare the peak inversion velocities between the different test situations, we have considered the potential overestimation during the material test, as shown in Figure 3.

When considering these adjustments, the mean inversion velocities observed for the sports-specific task in this study were lower than the velocities observed during the fast material testing situation. Furthermore, by comparing the measured inversion velocities against inversion velocities reported in the literature for actual injury situations (Figure 3),^10^ it is apparent that the material testing more closely resembles the real ankle strain injury mechanism than the biomechanical testing with real participants. Therefore, it may be concluded that the novel adaptive ankle brace provides a similar or slightly better protection against inversion-related ankle injuries in situations that resemble high injury risk.

The comparison of peak inversion velocities between testing conditions further highlights the need for material testing using artificial ankle joints or cadaveric preparations to understand the effects of adaptive technologies that change their behavior based on the intensity of movement. Using such methodological approaches allows for the testing of protection technologies in situations that have been shown to cause injuries. Overall, it appears that the adaptive ankle brace offers a similar amount of protection under high-risk, high–inversion velocity conditions.

Next to protecting against excessive ankle inversion, users of ankle protection systems still want to perform well in their respective sports when wearing a protection system. Sports performance was evaluated in the current study during sports-relevant motions. In these tasks, we observed no significant main effects of orthotic conditions except for CMJ. Here, the adaptive brace did not show a significant difference from the baseline condition (–1.7%), while the lace-up and rigid braces showed significantly lower CMJ heights (–3.8% and –2.7%, respectively, compared with baseline) than the adaptive brace. Overall, we concluded that differences between adaptive and passive ankle braces regarding sports performance are likely minor. However, future studies should evaluate these orthotic conditions in more realistic game/sports situations to strengthen the ecological validity of the test scenario.

When considering the freedom of movement during slower motions with low injury risk, the adaptive brace outperformed the passive brace conditions in this study. Active range of motion values were increased by 46% and 76% for the adaptive brace compared with the lace-up and rigid brace conditions, respectively. This result matched the subjective perception of feeling restricted. Here, the adaptive brace was subjectively rated as restricting the motion of the ankle joint clearly less than the passive brace conditions.

Furthermore, the participants rated the adaptive brace as more comfortable. Interestingly, the participants rated the adaptive product as providing a similar amount of stability to the passive braces. This subjective stability rating seems to be more in line with the findings from the material testing than the findings from the biomechanical testing of the protective effects of the products.

Whether the findings of the present study translate to better user compliance in real sports situations should be investigated in future prospective studies addressing injury incidences and compliance aspects. With neuromuscular training interventions and other technological interventions, for example, reducing lateral shoe traction,^9^ adaptive ankle protection might reduce ankle injury prevalence by offering protection with less restriction and better comfort for the users.

### Limitations

The findings of this study do not come without limitations. We included only male participants to improve the homogeneity of the participant sample. However, future studies must validate these findings for female participants. Furthermore, we mainly tested participants with no injuries (ie, healthy, intact ligaments). Athletes with a history of ankle injury or present injury (ie, those with functional or structural instability) may respond differently to these braces. In addition, we only tested the right leg of our participants, which was the dominant leg for most of them. Future studies need to verify our findings for the nondominant leg. Finally, the biomechanical tests performed in this study were performed in nonfatigued conditions. Because fatigue can alter the injury risk profile for lateral ankle injuries,^18^ its effects should be better integrated into future studies assessing ankle protection technologies.

## Conclusion

Overall, we found that the novel adaptive ankle brace offers similar protective effects in high-velocity inversion situations to those of passive protection technologies, while affecting sports performance–related tasks very little. At the same time, the adaptive brace improved active ankle range of motion, as well as subjective comfort and restriction ratings compared with the passive braces.

## Supplemental Material

sj-pdf-1-ajs-10.1177_03635465221146294 – Supplemental material for A Multidimensional Assessment of a Novel Adaptive Versus Traditional Passive Ankle Sprain Protection Systems Click here for additional data file. Supplemental material, sj-pdf-1-ajs-10.1177_03635465221146294 for A Multidimensional Assessment of a Novel Adaptive Versus Traditional Passive Ankle Sprain Protection Systems by Steffen Willwacher, Anna Bruder, Johanna Robbin, Jakob Kruppa and Patrick Mai in The American Journal of Sports Medicine
