# Supplementary material for: A Multidimensional Assessment of a Novel Adaptive Versus Traditional Passive Ankle Sprain Protection Systems
Source: Am J Sports Med. 2023 Feb 3;51(3):715–22. doi: 10.1177/03635465221146294 (PMC9983046; doi:10.1177/03635465221146294)
Supplement: sj-pdf-1-ajs-10.1177_03635465221146294 – Supplemental material for A Multidimensional Assessment of a Novel Adaptive Versus Traditional Passive Ankle Sprain Protection Systems [file sj-pdf-1-ajs-10.1177_03635465221146294.pdf]

APPENDIX

A multidimensional assessment of a novel adaptive versus traditional passive ankle sprain protection systems

Steffen Willwacher, Anna Bruder, Johanna Robbin, Jakob Kruppa, Patrick Mai

Table A1: Parameters of interest (mean ± sd) derived from the testing within participants (n = 20).

	Main Effect p-value	Partial η²	No Brace (1)	Adaptive (2)	Lace Up Brace (3)	Active Ankle (4)
**Protection:**						
Max. Inversion Tilt Platform (°)	<0.001	0.781	26.32 +- 2.41 [3,4]	25.28 +- 2.80 [3,4]	24.30 +- 3.06 [1,2,4]	19.20 +- 3.25 [1,2,3]
Max. Inversion Cut (°)	<0.001	0.566	24.56 ± 3.96 [4]	25.60 ± 4.92 [4]	24.57 ± 4.96 [4]	19.33 ± 4.79 [1,2,3]
Max. Inversion Sideshuffling (°)	<0.001	0.660	25.77 ± 5.44 [3,4]	27.26 ± 5.80 [4]	27.92 ± 5.68 [1,4]	20.16 ± 6.84 [1,2,3]
Avg. Hor. GRF Sideshuffling (N/k	0.555	0.036	6.74 ± 1.28	6.68 ± 1.29	6.64 ± 1.28	6.74 ± 1.43
Stiffness Material Test Slow (Nm/°)			[na]	0.04 ± 0.00 [na]	0.19 ± 0.00 [na]	0.20 ± 0.01 [na]
Stiffness Material Test Fast (Nm/°)			[na]	0.20 ± 0.01 [na]	0.19 ± 0.02 [na]	0.19 ± 0.00 [na]
**Sports performance:**						
CMJ Height (m)	0.016	0.165	0.45 ± 0.05 [3,4]	0.45 ± 0.06	0.44 ± 0.06 [1]	0.44 ± 0.05 [1]
Avg. Lin. Acceleration (m/s²)	0.194	0.079	4.42 ± 0.42	4.30 ± 0.43	4.26 ± 0.39	4.37 ± 0.36
Entry Velocity Cut (m/s)	0.654	0.028	3.49 ± 0.29	3.48 ± 0.27	3.45 ± 0.27	3.46 ± 0.29
Exit Velocity Cut (m/s)	0.527	0.038	2.88 ± 0.49	2.77 ± 0.65	2.77 ± 0.66	2.89 ± 0.45
Actual Change of Direction Cut (°	0.524	0.030	125.9 ± 11.0	124.8 ± 17.2	126.4 ± 13.0	129.0 ± 8.9
Contact Time Cut (s)	0.219	0.074	0.38 ± 0.07	0.37 ± 0.06	0.38 ± 0.06	0.37 ± 0.06
Side Hop Time (s)	0.391	0.051	3.01 ± 0.46	2.90 ± 0.54	3.01 ± 0.55	3.00 ± 0.39
**Freedom of movement:**						
RoM Slow Movement (°)	<0.001	0.812	31.79 ± 6.59 [2,3,4]	25.30 ± 5.64 [1,3,4]	17.22 ± 5.62 [1,2,4]	14.36 ± 3.17 [1,2,3]
**Subjective perceptiion:**						
Comfort Rating (VAS)	<0.001	0.526	9.00 ± 1.56 [2,3,4]	6.93 ± 2.70 [1,3,4]	5.37 ± 2.63 [1,2,4]	4.12 ± 2.95 [1,2,3]
Stability Rating (VAS)	0.003	0.284	4.41 ± 2.95 [2,3,4]	6.75 ± 2.57 [1]	7.09 ± 2.31 [1]	6.87 ± 2.48 [1]
Restriction Rating (VAS)	<0.001	0.637	9.38 ± 0.71 [2,3,4]	6.61 ± 2.34 [1,3,4]	4.48 ± 2.33 [1,2,4]	3.26 ± 2.95 [1,2,3]

[1,2,3,4] refers to a significant difference (t-test, p<0.05) compared to no brace (1), adaptive (2), lace up (3) or Active Ankle (4) brace, respectively.

[na] refers to the material tests where a statistical comparison of the results was not applicable (na).

Maximum ankle inversion angles were determined during the stance phase of each analyzed motion.

Avg. Hor. GRF describes the average mediolateral ground reaction force applied during the stance phase as an indicator of sideshuffling intensity.

CMJ Height refers to the jump height achieved during a two-legged countermovement jump.

Avg. Lin. Acceleration refers to the average center of mass acceleration during the first ground contact after a standing start with maximal effort.

Entry and exit velocity refer to the linear velocity of the center of mass (estimated by the midpint of the pelvis markers) before and after contacting the ground, respectively.

Actual change of direction refers to the angle between the center of mass motion before and after contacting the ground during the cutting maneuver.

Side Hop Time is the time needed to complete 5 single leg sidewards hops over a distance of 0.3 m.

RoM Slow Movement was determined as the maximum range of motion of the ankle in the frontal plane of motion during a slow self initiated ankle movement.

For comfort and stability ratings, higher VAS values represent a more comfortable or more stable condition, while for restriction ratings, a higher value refers to less restriction.
